# Depletion of MGO or Its Derivatives Ameliorate CUMS-Induced Neuroinflammation

**DOI:** 10.3390/cells14060397

**Published:** 2025-03-08

**Authors:** Bing Liu, Ke Dong, Yun Zhao, Xue Wang, Zhaowei Sun, Fang Xie, Lingjia Qian

**Affiliations:** 1 Beijing Institute of Basic Medical Sciences, Academy of Military Medical Sciences, Beijing 100850, China; liubingzsp@163.com (B.L.); dongke0302@163.com (K.D.); 15710282409@139.com (Y.Z.); snowwang0326@foxmail.com (X.W.); sunzhw0820@163.com (Z.S.); 2 School of Medicine, South China University of Technology, Guangzhou 511442, China

**Keywords:** CUMS, neuroinflammation, microglia, MGO, RAGE

## Abstract

Advanced glycation end products (AGEs) are a series of structurally complex and harmful compounds formed through the reaction between the carbonyl group of reducing sugars (such as glucose and fructose) and the free amino groups of proteins, lipids, or nucleic acids. Excessive accumulation of AGEs in the body can trigger oxidative stress, induce inflammatory responses, and contribute to the development of diabetes, atherosclerosis, and neurological disorders. Within the category of dicarbonyl compounds, methylglyoxal (MGO)—a byproduct resulting from glucose degradation—serves as a pivotal precursor in the formation of AGEs and the induction of neurotoxicity. Specifically, AGEs generated from MGO display significant cytotoxicity toward cells in the central nervous system. Therefore, we aimed to investigate the role of MGO-AGEs in neuroinflammation mediated by CUMS. Interestingly, we found that the overexpression of glyoxalase 1 (GLO1) reduced the levels of MGO in corticosterone-treated microglia, thereby alleviating the inflammatory response. Furthermore, overexpression of GLO1 in the hippocampus of chronically stressed mice reduced MGO levels, mitigating CUMS-induced neuroinflammation and cognitive impairment. Additionally, when using the receptor for advanced glycation end products (RAGE) inhibitor FPS-ZM1 in primary microglia cells, we observed that despite corticosterone-induced elevation of MGO, no significant inflammatory response occurred. This suggests that RAGE clearance can reduce MGO-AGE-mediated neurotoxicity. Subsequently, we used FPS-ZM1 to treat chronically stressed mice and found that it significantly ameliorated neuroinflammation and cognitive dysfunction. These results suggest that targeting MGO metabolism could serve as a therapeutic approach to manage neuroinflammation in stress-related mental disorders.

## 1. Introduction

The Chronic Unpredictable Mild Stress (CUMS) paradigm is a widely employed animal model designed to emulate chronic stress conditions observed in humans. Sustained exposure to CUMS induces metabolic disturbances, cognitive impairments, and behavioral alterations, all of which are implicated in the pathophysiology of mental disorders such as depression and anxiety [1,2,3,4]. On the physiological and biochemical levels, CUMS not only triggers hyperactivation of the hypothalamic–pituitary–adrenal (HPA) axis but is also closely linked to oxidative stress, inflammatory responses, and metabolic abnormalities [5,6,7,8]. Among these metabolic disturbances, the generation of methylglyoxal (MGO) and advanced glycation end products (AGEs) is considered to be significant pathogenic factors [9,10].

MGO is a highly reactive small-molecule metabolic intermediate, primarily generated during glycolysis, and can accumulate through endogenous or exogenous pathways. MGO, due to its high reactivity, can undergo glycation, autoxidation, or lipid peroxidation with amino acid residues of macromolecules such as proteins and nucleic acids, forming AGEs through the Maillard reaction [9,11]. The accumulated AGEs bind to their receptors, activating multiple inflammatory signaling pathways, inducing cellular damage and inflammation, leading to tissue dysfunction, and contributing to the onset of chronic diseases such as diabetic complications, cardiovascular diseases, and neurodegenerative disorders. Given MGO’s central role in glucose metabolism, its involvement in AGE formation has made it a key area of research in metabolic disorders, though its role in CUMS-induced cognitive impairment has yet to be fully elucidated [12,13,14,15,16].

Growing evidence suggests that AGEs are implicated in a range of diseases, including neurological complications, psoriasis, metabolic disorders, diabetes, and vascular complications [17]. While earlier studies primarily focused on the harmful effects of AGEs in diabetes, recent findings reveal a strong association between AGEs and CUMS-induced neuroinflammation, hippocampal neuronal damage, and cognitive decline. Research has shown that the accumulation of AGEs not only impairs neuronal function but also disrupts the blood–brain barrier, exacerbating inflammation in brain tissues and worsening stress-induced behavioral abnormalities [18,19,20]. In brain cells, particularly microglia and infiltrating macrophages, the interaction between AGEs and their receptor (RAGE) drives their activation toward a pro-inflammatory phenotype, leading to excessive production of pro-inflammatory mediators. The results indicate that MGO and AGEs might be crucial factors in stress-induced psychiatric disorders. Inhibiting MGO formation, enhancing AGE breakdown, or interrupting the interaction between AGE and RAGE may be promising approaches for treating chronic stress-related conditions [16,21].

Glyoxalase 1 (GLO1) is a key enzyme responsible for detoxifying MGO produced during metabolism. By catalyzing the reaction between MGO and glutathione to form S-lactoylglutathione, GLO1 reduces MGO accumulation and protects cells from its cytotoxic effects. The proper function of GLO1 is crucial for maintaining intracellular metabolic balance and reducing AGE formation. Insufficient GLO1 expression or reduced activity has been linked to several diseases, including diabetes, Alzheimer’s disease, and depression [22,23]. FPS-ZM1, a potent and specific RAGE inhibitor, blocks the binding of AGEs to the RAGE, thereby preventing the activation of inflammatory signaling pathways that lead to oxidative stress and inflammatory responses. FPS-ZM1 has demonstrated neuroprotective effects in various stress-related disease models, ameliorating cognitive function and reducing neuroinflammation, suggesting its potential therapeutic value in treating AGE accumulation-related diseases such as Alzheimer’s disease, diabetic complications, and chronic stress-induced psychiatric disorders. In this study, we investigated whether GLO1 and FPS-ZM1 could serve as potential candidates for mitigating MGO-AGE-mediated neuroinflammation [24,25,26].

## 2. Materials and Methods

### 2.1. Animals Experiment

Male C57BL/6 wild-type mice, aged 8 weeks, were procured from HuaFuKang Company (Beijing, China) and subsequently assigned randomly to distinct experimental cohorts. The Institutional Animal Care and Use Committee of the Academy of Military Medicine Sciences (Permit No: IACUC-DWZX-2022-534) approved all animal experiments, which were conducted in accordance with the National Research Council’s Guide for the Care and Use of Laboratory Animals (8th edition). Mice were maintained in environmentally controlled conditions and provided with ad libitum access to standard rodent chow and water, except during periods of stress exposure and behavioral assessment. Following the completion of behavioral experiments, mice were anesthetized intraperitoneally with Tribromoethanol (Meilunbio, MA0478) at a dosage of 30 μL/g body weight, and brain tissues were harvested for subsequent analyses.

### 2.2. Chronic Unpredictable Mild Stress (CUMS)

Following a 7-day acclimation period to the housing environment, the mice were exposed to chronic unpredictable mild stress (CUMS) as previously described [27,28]. In brief, two random stressors were given daily, including 24 h fasting, 24 h water deprivation, 12 h wet bedding, 24 h cage tilting at a 30° angle, 6 h physical restraint, 2 min forced swimming in 4 °C water, 30 min shaking at 120 rpm, and overnight bright light exposure. The control group, however, was maintained under standard housing conditions.

### 2.3. Cell Culture and Treatment

The BV2 mouse microglia cell line was obtained from the American Type Culture Collection (ATCC). The cells were cultured in Dulbecco’s Modified Eagle Medium (DMEM, Gibco) supplemented with 10% fetal bovine serum (FBS, 10099141C, Gibco, Grand Island, NY, USA) and penicillin–streptomycin (100 U/mL, 15140122, Gibco). Cultures were maintained in a humidified incubator at 37 °C with 5% CO_2_. The cells were exposed to various concentrations of corticosterone (MedChemExpress, New Jersey, NJ, USA) for 24 h.

### 2.4. Primary Culture of Microglia

Cells were dissociated and seeded into 75 cm^2^ flasks, followed by cultivation in minimum essential medium (DMEM/F-12, Gibco) supplemented with 10% fetal bovine serum (Gibco) and 1% penicillin–streptomycin (100 U/mL, Gibco) at 37 °C. After a culture period of 12–14 days, mature microglia were isolated from mixed glial cultures via gentle, rapid shaking. The isolated microglia were subsequently resuspended in F12 medium containing 10% FBS and transferred to new plates. The cells were permitted to adhere for 2–3 h prior to proceeding with further experiments [29].

### 2.5. FPS-ZM1 Treatment

FPS-ZM1 (HY-19370, MedChemExpress, New Jersey, NJ, USA) was used as an inhibitor of RAGE to investigate the potential role of high RAGE response in cognitive decline induced by CUMS. After being dissolved in DMSO and diluted to the appropriate concentrations, FPS-ZM1 (1 μM) was directly added to the culture medium 1 h prior to corticosterone treatment for cell experiments. For animal experiments, FPS-ZM1 (1 mg/kg) was administered via intraperitoneal injection once every two days during the last 4 weeks of the CUMS procedure (weeks 5–8). The same volume of DMSO (2% for animals) was used as the vehicle control. According to the manufacturer’s instructions, the inhibitor can cross the blood–brain barrier, and the doses used in this study were based on published studies [25].

### 2.6. Morris Water Maze (MWM)

The Morris water maze test was employed to evaluate spatial learning and memory in mice, following a modified version of a previously established protocol [30]. The assessment was performed in a circular water maze with a diameter of 1.2 m, maintained at a water temperature ranging from 19 to 22 °C. The experimental procedure was conducted in a quiet laboratory setting from isolated external auditory distractions. Mice were subjected to daily training sessions, each lasting 3 min, for a total of 5 consecutive days. During each session, mice were allotted 180 s to swim freely and locate the submerged platform. In instances where a mouse failed to find the platform within the allotted time, it was gently guided to the platform and permitted to remain there for 30 s. The time required to reach the hidden platform, termed escape latency, was meticulously recorded. In the testing phase, the platform was removed, and the mice were placed in the water facing the wall opposite the quadrant where the platform had been. The time taken to reach the platform’s former location and the number of times the mice crossed the platform area during a 3 min period were recorded. Movements were tracked using the Noldus EthoVision XT 16 system from Noldus Information Technology BV, Wageningen, The Netherlands.

### 2.7. Novel Object Recognition Test

The novel object recognition test evaluates memory in mice [31]. Mice were positioned near the center of the front wall, facing away from two objects, in an arena (50 cm × 50 cm × 50 cm) for 5 min to perform a recognition task involving two identical objects in terms of color, size, shape, and weight (heavy enough to prevent movement by the animals). This initial test ensured no inherent preferences or aversions to the objects and equal exploration durations for both objects (habituation phase). To assess short-term memory, the mice were reintroduced to the arena 30 min later with one familiar and one novel object (differing in shape and size). Exploration was defined as directing the nose within 2 cm of the object or touching it with the nose or mouth. Each 5 min session was videotaped and analyzed. The recognition index was calculated as the proportion of time spent exploring the novel object (N) relative to the total exploration time for both objects (N + F) = N / (N + F) × 100%.

### 2.8. Enzyme-Linked Immunosorbent Assay (ELISA)

The hippocampal tissues were removed from the CUMS and the Control mice, and prechilled PBS was added. The sample mixture was homogenized using a grinder. After centrifugation at 1000× *g* for 10 min at 4 °C, the hippocampal tissue supernatant was processed with Mouse Corticosterone ELISA kit and NA/NE ELISA kit (Sangon, Shanghai, China). Similarly, hippocampal tissue supernatants were analyzed using mouse IL-6, IL-1β, and TNF-α ELISA kits (ABclonal, Wuhan, China), MGO and AGEs ELISA kits (E03M0040, E03A0002, Bluegene, Shanghai, China), RAGE ELISA kits (CSB-EL001441MO, CUSABIO, https://www.cusabio.com/, 3 January 2025). The collected, cultured medium from BV2 cells and primary microglia cells was centrifugated at 1000× *g* for 15 min at 4 °C, and the supernatant was analyzed using Mouse IL-6, IL-1β, TNF-α, MGO, RAGE, AGEs ELISA kits, following the manufacturer’s instructions [32].

### 2.9. Glucose Measurement of Hippocampus

Hippocampal tissue was collected and weighed from the brains of CUMS and Control mice. Then, we added 1 mL of distilled water per 0.1 g of tissue and homogenized it with an electric homogenizer. The mixture was incubated in a 95 °C water bath for 10 min and then centrifuged at 8000× *g* for 10 min. We collected the supernatant and analyzed it using a Glucose Content Assay kit (Sangon Biotech, Shanghai, China) according to the manufacturer’s instructions [32].

### 2.10. Immunofluorescence (IF) Staining

For immnohistofluorescence, the brains were separated and post-fixed in 4% paraformaldehyde (PFA) at 4 °C overnight. The fixed brains were dehydrated in 10–30% (*w*/*v*) sucrose for cryoprotection and embedded in Tissue-Tek O.C.T. compound (#4583, SAKURA, Torrance, CA, USA). The brain samples were cut into 25 µm thick sections using a Leica CM1950 cryostat and subjected to immunohistofluorescence following routine protocols as described previously [33]. GFP (1:200, Abcam, Cambridge, MA, USA, ab13970), IBA1(1:200, Abcam, USA, ab283319), and RAGE (1:200, Abcam, USA, ab37647) were used for staining. The secondary antibodies included Goat Anti-Mouse IgG H&L (Alexa Fluor^®^ 488) (1:200, Abcam, USA, ab150113), Goat Anti-Chicken IgG H&L (Alexa Fluor^®^ 488) (1:200, Abcam, USA, ab150173), and Goat anti-Rabbit IgG H&L (Alexa Fluor^®^ 594) (1:200, Abcam, USA, ab150080). Cell nuclei were stained with 4′,6-diamino-2-phenylindole (DAPI) (Sigma Aldrich, St. Louis, MO, USA). Images were captured using a confocal laser scanning microscope (Olympus, Tokyo, Japan).

### 2.11. Quantitative Real-Time PCR (qRT-PCR)

Total RNA was isolated from microglia cells and mouse hippocampal tissues using TRIzol reagent (Sigma) and subsequently reverse transcribed into cDNA using the RT Master Mix (#G490, Abmart). Quantitative real-time PCR (qRT-PCR) was then conducted on a LightCycler 96 Real-time PCR System (Roche, Basel, Switzerland) with the TB Green Pre-mix Ex Taq kit (TaKaRa, Kyoto, Japan). β-Actin was used as an endogenous control. The relative expression levels of the target genes were determined using the 2^−ΔΔCt^ method [34]. The sequences of the primers used are listed in Supplementary Table S1.

### 2.12. Western Blotting

Fresh hippocampus tissues or cells were sufficiently lysed with RIPA buffer supplemented with a protease inhibitor cocktail (MedChemExpress, Monmouth Junction, NJ, USA) and subsequently centrifuged at 12,000× *g* for 20 min at 4 °C to extract the supernatant. Twenty-five micrograms (25 μg) of protein extracts were loaded onto 8–12% SDS-PAGE. The resolved proteins were transferred onto PVDF membranes and blocked with 5% non-fat dry milk in TBST buffer at room temperature for 1 h. The membranes were probed with primary antibodies against MGO (1:1000, Cat# STA-011, Cell Biolabs, San Diego, CA, USA), RAGE (1:1000, Cat# ab37647, Abcam), GLO1 (1:1,000, Cat# ab137098, Abcam), β-actin (1:1000, 66009-1-Ig, ProteinTech, Rosemont, IL, USA) and incubated overnight at 4 °C. Afterwards, the membranes were probed with HRP-conjugated secondary antibody (1:5000, R&D Systems, Abingdon, UK) for 1 h at room temperature. The membranes were processed using the ECL Western blotting substrate kit (Thermo Fisher Scientific, Waltham, MA, USA), followed by image acquisition on the ImageQuant LAS 4000 system (GE Healthcare, Bronx, NY, USA) and subsequent analysis using ImageJ software, Version 1.54p, RRID:SCR_003070, NIH, USA.

### 2.13. RNA Interference

BV2 cells were transfected with a lentivirus encoding GLO1-shRNA for 72 h, followed by selection with puromycin (Sigma, St. Louis, MO, USA). Single-cell clones that were resistant to puromycin were expanded and further screened by Western blot using an anti-GLO1 antibody (1:1000, Cat# ab137098, Abcam). The lentivirus was produced by Genechem (Shanghai, China) [34].

### 2.14. Virus

All the viruses used in our study were produced by Genechem. (Shanghai, China): adeno-associated virus (AAV)-F4/80p-GLO1-EGFP-3Flag-SV40-PolyA, AAV-F4/80p-Vector-EGFP-3Flag-SV40-PolyA. The viruses were bilaterally injected into the hippocampus (coordinates: −2.2 mm caudal to bregma, ±1.9 mm from midline, −1.9 mm deep from the skull) by stereotactic localization at an AAV dose of 3 × 10^9^ vg [28].

### 2.15. Statistical Analysis

The GraphPad Prism 8.0 was used, and data were presented as mean ± SD. Normality of continuous variables was evaluated. To assess the significance of differences between two independent groups, a two-tailed unpaired Student’s *t*-test was used. One-way ANOVA was used for the comparisons of more than three groups, followed by Tukey’s multiple comparisons test. *p* < 0.05 was considered to be significant.

## 3. Results

### 3.1. CUMS Induces Cognitive Decline and Activates Neuroinflammation in Mice

**Figure 1:**
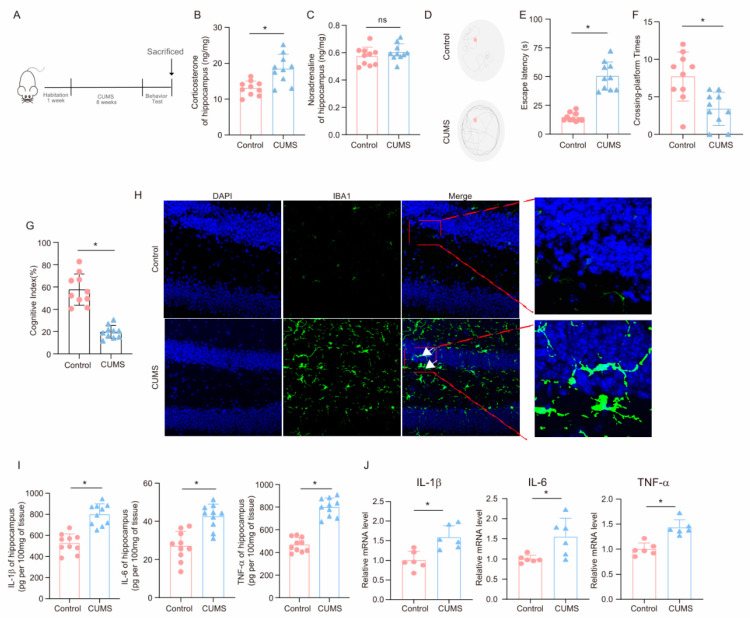
**CUMS induces cognitive decline and activates neuroinflammation in mice.** (**A**) Schematic illustration of the experimental timeline for CUMS and behavioral testing. (**B**) Hippocampal corticosterone concentrations (ng/mg) in Control and CUMS mice at the conclusion of the CUMS protocol (*n* = 10; Student’s *t*-test, * *p* < 0.05). (**C**) Concentrations (ng/mg) of norepinephrine in the hippocampus tissues of Control and CUMS mice at the end of the CUMS procedure (*n* = 10, Student’s *t*-test, ns: no significant). (**D**) Representative track images of mice in the probe trial of MWM. (**E**,**F**) Escaping latency and Crossing-platform Times of mice (*n* = 10, Student’s *t*-test, * *p* < 0.05). (**G**) Cognitive Index of mice (*n* = 10, Student’s *t*-test, * *p* < 0.05). (**H**) Representative images of IF staining of hippocampal sections from Control and CUMS mice. Iba-1, green; DAPI, blue. Scale bar, 50 μm. (**I**) Levels of IL-1β, IL-6, and TNF-α in hippocampus lysates from Control and CUMS mice as determined by ELISA (*n* = 10, Student’s *t*-test, * *p* < 0.05). (**J**) The mRNA level for the IL-1β, IL-6, and TNF-α in hippocampus lysates from Control and CUMS mice (*n* = 6, Student’s *t*-test, * *p* < 0.05).

After the completion of the 8-week CUMS program (Figure 1A), key hormones and cognitive behaviors were measured to evaluate the effects of CUMS on the brain. As shown in Figure 1B,C, corticosterone levels in the hippocampus of CUMS mice were elevated compared to those of Control mice, while norepinephrine levels remained unchanged, aligning with the idea that glucocorticoids are the primary agents in chronic stress-related damage [35]. Notably, in the MWM test, CUMS mice required more time to locate the platform than Control mice (Figure 1D,E). Additionally, during the test phase, CUMS mice crossed the platform fewer times than Control mice (Figure 1F). Moreover, CUMS mice demonstrated a reduced cognitive index (Figure 1G). These findings indicate that CUMS may lead to cognitive decline in mice.

The hippocampus plays a crucial role in spatial learning and memory. To examine the effects of CUMS on microglia in the hippocampus, we collected hippocampal tissues at the end of the CUMS program. Immunofluorescence analysis employing the microglia marker Iba-1 demonstrated that CUMS induced microglia activation (Figure 1H). Furthermore, we explored the influence of CUMS on inflammatory factors in the hippocampus. ELISA results showed significant increases in pro-inflammatory factors, including IL-1β, IL-6, and TNF-α, in the hippocampus of CUMS mice (Figure 1I). Additionally, the mRNA expression levels of these inflammatory factors were elevated in CUMS mice (Figure 1J). In conclusion, these findings suggest that CUMS leads to cognitive decline in mice and activates neuroinflammation.

### 3.2. CUMS Induces the High Level of MGO and Its Derivatives in the Mouse Hippocampus

**Figure 2:**
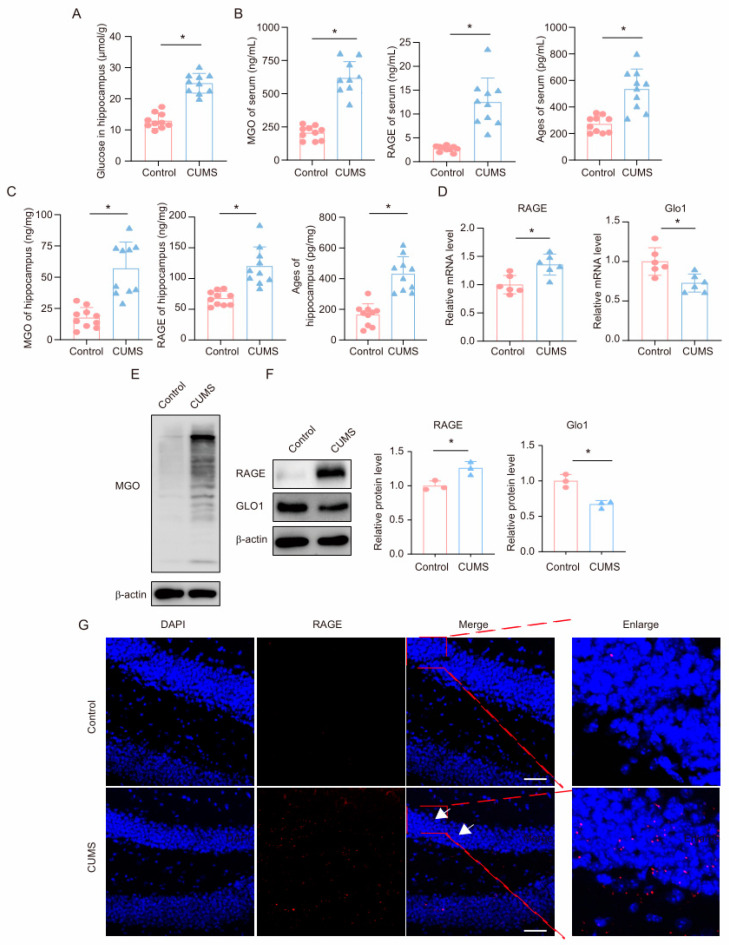
**CUMS induces a high level of MGO and its derivatives in the mouse hippocampus.** (**A**) Glucose levels in hippocampus lysates from Control and CUMS mice (*n* = 10, Student’s *t*-test, * *p* < 0.05). (**B**) Concentrations of MGO, RAGE, and Ages in the serum of Control and CUMS mice after the end of the CUMS procedure (*n* = 10, Student’s *t*-test, * *p* < 0.05). (**C**) Concentrations of MGO, RAGE, and Ages in the hippocampus of mice (*n* = 10, Student’s *t*-test, * *p* < 0.05). (**D**) qRT-PCR assays monitoring expression levels of RAGE and GLO1 in hippocampal samples from Control and CUMS mice (*n* = 6, Student’s *t*-test, * *p* < 0.05). (**E**) The MGO protein level in hippocampus lysates from mice. (**F**) The protein level of RAGE and GLO1 in hippocampus lysates from mice (*n* = 3, Student’s *t*-test, * *p* < 0.05). (**G**) Representative IF staining images of hippocampal sections from mice. RAGE, red; DAPI, blue. Scale bar, 50 μm.

First, we measured glucose levels in the hippocampus of mice. Consistent with previous reports, we found that glucose levels were significantly upregulated in the hippocampus of CUMS mice (Figure 2A). We subsequently measured the levels of the glycolysis intermediate MGO and its derivatives in the serum and hippocampal tissues of mice using ELISA. The results showed that, compared to the control group, CUMS mice exhibited significantly increased levels of MGO, RAGE, and AGEs in both the serum and hippocampal tissues (Figure 2B,C). Additionally, we measured MGO levels in the hippocampus using Western blot, revealing a marked increase in MGO levels in the hippocampus of CUMS mice (Figure 2E). GLO1 is an enzyme that detoxifies harmful by-products of glycolysis, particularly MGO. MGO can react with RAGE by forming AGEs, contributing to processes such as inflammation, oxidative stress, and cell migration. We examined the expression levels of GLO1 and RAGE in the hippocampus of mice and found that in the CUMS model, both mRNA and protein expression levels of GLO1 were decreased, while RAGE expression was significantly increased (Figure 2D,F). Moreover, immunofluorescence results indicated an increased RAGE presence in the hippocampus (Figure 2G). These findings demonstrate that during chronic stress, levels of the glycolysis intermediate MGO and its derivatives increase in the hippocampus of mice.

### 3.3. Corticosterone Induces Neuroinflammation in Microglia and the High Level of MGO and Its Derivatives

**Figure 3:**
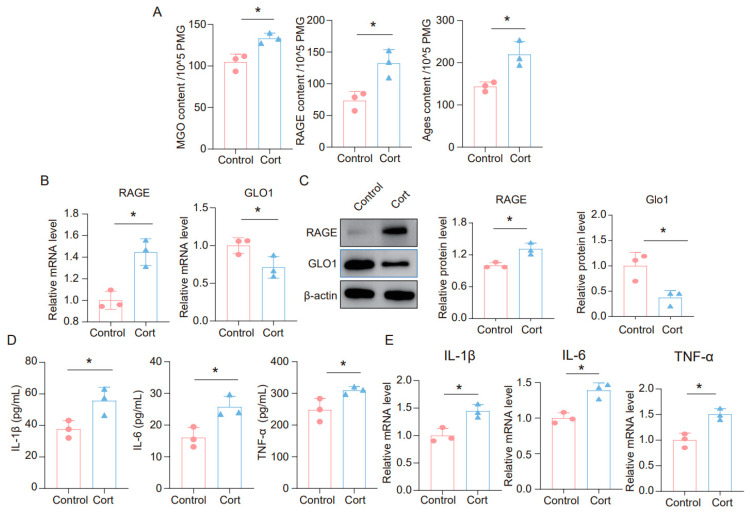
**Corticosterone induces neuroinflammation in primary microglia and the high level of MGO and its derivatives.** (**A**) Concentrations of MGO, RAGE, and Ages in primary microglia cells from Control and Cort as determined by ELISA (*n* = 3, Student’s *t*-test, * *p* < 0.05). (**B**) qRT-PCR assays monitoring expression levels of RAGE and GLO1 in primary microglia cells (*n* = 3, Student’s *t*-test, * *p* < 0.05). (**C**) The protein level of RAGE and GLO1 in primary microglia cells (*n* = 3, Student’s *t*-test, * *p* < 0.05). (**D**) Concentrations of IL-1β, IL-6, and TNF-α in primary microglial cells as quantified by ELISA. (*n* = 3, Student’s *t*-test, * *p* < 0.05). (**E**) The mRNA level of IL-1β, IL-6, and TNF-α in primary microglia cells (*n* = 3, Student’s *t*-test, * *p* < 0.05).

It is widely believed that glucocorticoids (GC) are a key contributor to chronic stress-related damage. To explore the impact of corticosterone on neuroinflammatory responses in microglia, as well as on MGO and its derivatives, we exposed BV2 and primary microglia cells to corticosterone. The findings revealed that corticosterone significantly elevated the levels of MGO, RAGE, and AGEs in microglia cells (Figure 3A and Figure S1A). Furthermore, we analyzed the mRNA levels of RAGE and GLO1 in these cells and discovered that corticosterone caused an upregulation of RAGE and a downregulation of GLO1 (Figure 3B and Figure S1B). We also assessed the protein expression of RAGE and GLO1 in primary microglia cells, where corticosterone was found to promote increased RAGE and decreased GLO1 expression at the protein level (Figure 3C). Additionally, ELISA results demonstrated that the levels of inflammatory factors such as IL-1β, IL-6, and TNF-α were significantly raised in BV2 and primary microglia cells treated with corticosterone (Figure 3D and Figure S1C). Similarly, qPCR data indicated that corticosterone stimulated an increase in the expression of inflammatory factors in microglia cells (Figure 3E and Figure S1D). Collectively, these results suggest that exposure to corticosterone triggers an inflammatory response in microglia cells and leads to an accumulation of MGO and its derivatives.

### 3.4. MGO Depletion Ameliorates Corticosterone-Induced Neuroinflammation in BV2 Cells

**Figure 4:**
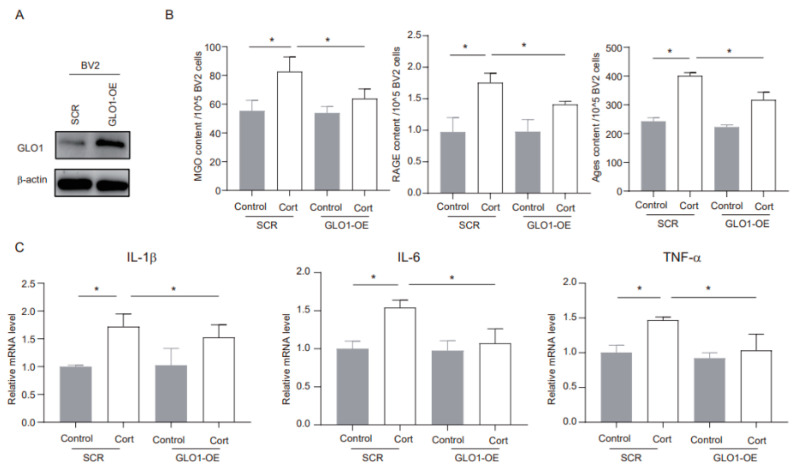
**MGO depletion ameliorates corticosterone-induced neuroinflammation in BV2 cells.** (**A**) The overexpression efficacy was investigated using Western blotting. (**B**) Concentrations of MGO, RAGE, and Ages in BV2 cells as determined by ELISA (*n* = 3, One-way ANOVA with Tukey’s post hoc test, * *p* < 0.05). (**C**) Levels of IL-1β, IL-6, and TNF-α in BV2 cells as determined by ELISA (*n* = 3, One-way ANOVA with Tukey’s post hoc test, * *p* < 0.05).

GLO1 is an enzyme that detoxifies harmful byproducts of glycolysis, particularly MGO [22]. We utilized shRNA to overexpress endogenous GLO1 and evaluated its potential role in corticosterone-mediated microglia inflammation. The overexpression method successfully increased GLO1 protein expression (Figure 4A). Furthermore, corticosterone exposure significantly elevated the levels of MGO and its derivatives in negative control (SCR) cells, while in GLO1-overexpressing (GLO1-OE) cells, these levels were notably reduced (Figure 4B). In addition, GLO1 overexpression significantly inhibited the corticosterone-induced increase in inflammatory markers (Figure 4C). Overall, these findings indicate that GLO1 may play a protective role in corticosterone-induced neuroinflammation by reducing the levels of MGO and its derivatives.

### 3.5. FPS-ZM1 Ameliorates Corticosterone-Induced Microglia Inflammation and MGO and Its Derivatives

**Figure 5:**
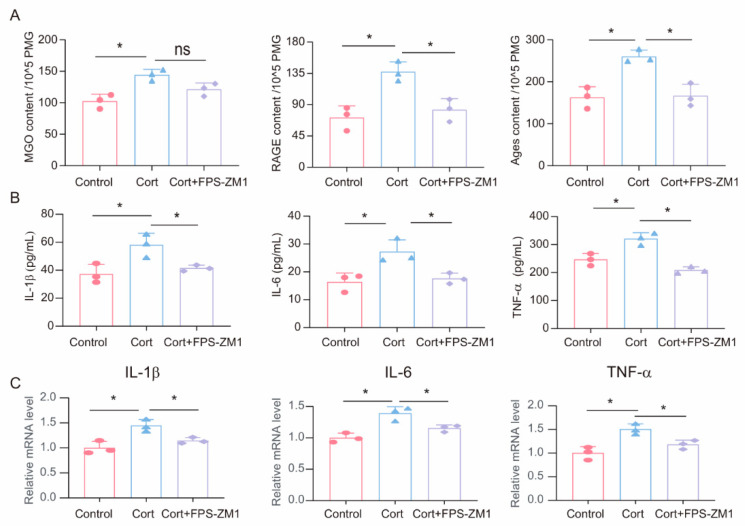
**FPS-ZM1 ameliorates corticosterone-induced microglia inflammation and MGO and its derivatives.** (**A**) Concentrations of MGO, RAGE, and Ages in primary microglia cells from Control, Cort, and Cort+FPS-ZM1 as determined by ELISA (*n* = 3, One-way ANOVA with Tukey’s post hoc test, * *p* < 0.05, ns: no significant). (**B**) Levels of IL-1β, IL-6, and TNF-α in primary microglia cells from Control, Cort, and Cort+FPS-ZM1 as determined by ELISA (*n* = 3, One-way ANOVA with Tukey’s post hoc test, * *p* < 0.05). (**C**) qRT-PCR assays monitoring the expression of inflammatory factors IL-1β, IL-6, and TNF-α in primary microglia cells from Control, Cort, and Cort+FPS-ZM1 (*n* = 3, One-way ANOVA with Tukey’s post hoc test, * *p* < 0.05).

To investigate the potential role of MGO-derived AGEs and RAGE in corticosterone-induced microglia inflammatory responses, we treated primary microglia cells with a combination of the RAGE inhibitor FPS-ZM1 (1 μM) and corticosterone (50 μM). First, we measured the levels of MGO, AGEs, and RAGE in primary microglia cells following corticosterone exposure. ELISA results showed that while the RAGE inhibitor did not significantly affect corticosterone-induced MGO levels, it reduced intracellular AGEs and RAGE levels (Figure 5A). Moreover, ELISA and real-time quantitative PCR results indicated that FPS-ZM1 treatment decreased the levels and mRNA expression of inflammatory markers in primary microglia (Figure 5B,C). Based on these results, the RAGE inhibitor FPS-ZM1 can attenuate corticosterone-induced microglia inflammation by inhibiting the cross-linking between AGEs and RAGE.

### 3.6. GLO1-Specific Overexpression of Hippocampal Microglia Ameliorates CUMS-Induced Cognitive Impairment, Inflammatory Response, and Production of MGO and Its Derivatives

**Figure 6:**
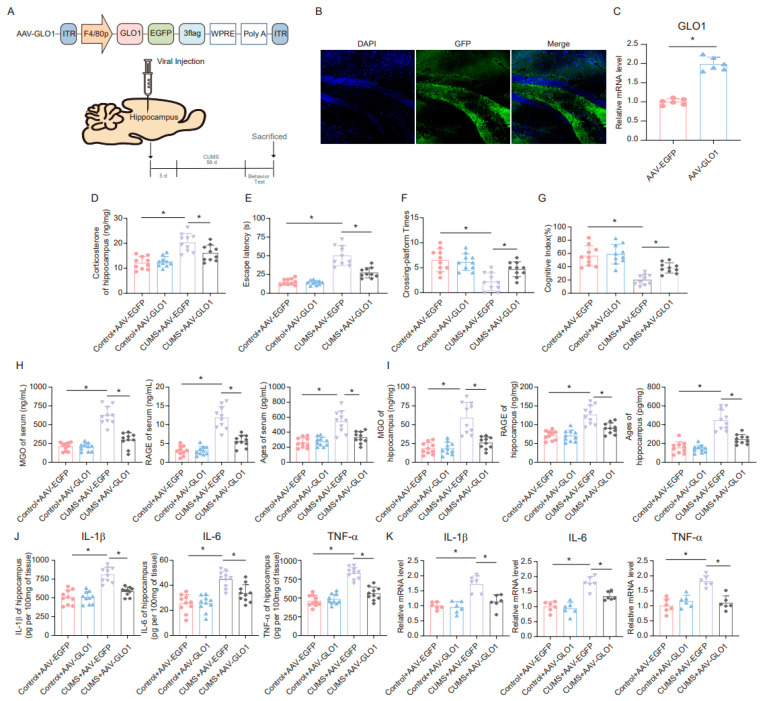
**GLO1-specific overexpression of hippocampal microglia ameliorates CUMS-induced cognitive impairment, inflammatory response, and production of MGO and its derivatives.** (**A**) Schematics of the AAV construct expressing GLO1-targeted specifically in microglia (AAV-GLO1) (upper). AAV-GLO1 or AAV-Control was injected into the mouse hippocampus, and the experimental timeline is shown (lower). (**B**) Representative fluorescence image of GFP in the mouse hippocampus after AAV-GLO1 infection. Scale bar, 100 μm. (**C**) qRT-PCR assay monitoring the expression of GLO1 in the mouse hippocampus injected with AAV-GLO1 or AAV-Control (*n* = 6, Student’s *t*-test, * *p* < 0.05). (**D**) Concentrations of corticosterone in the hippocampus of Control and CUMS mice after AAV-GLO1 or AAV-Control infection at the end of the CUMS procedure (*n* = 10, One-way ANOVA with Tukey’s post hoc test, * *p* < 0.05). (**E**,**F**) Escaping latency and Crossing-platform Times of Control and CUMS mice after AAV-GLO1 or AAV-Control infection at the end of the CUMS procedure (*n* = 10, One-way ANOVA with Tukey’s post hoc test, * *p* < 0.05). (**G**) Cognitive Index of Control and CUMS mice after AAV-GLO1 or AAV-Control infection at the end of the CUMS procedure (*n* = 10, One-way ANOVA with Tukey’s post hoc test, * *p* < 0.05). (**H**) Concentrations of MGO, RAGE, and Ages in the serum of Control and CUMS mice after AAV-GLO1 or AAV-Control infection at the end of the CUMS procedure (*n* = 10, One-way ANOVA with Tukey’s post hoc test, * *p* < 0.05). (**I**) Concentrations of MGO, RAGE, and Ages in the hippocampus of Control and CUMS mice after AAV-GLO1 or AAV-Control infection (*n* = 10, One-way ANOVA with Tukey’s post hoc test, * *p* < 0.05). (**J**) Levels of IL-1β, IL-6, and TNF-α in hippocampus lysates from Control and CUMS mice infected with AAV-GLO1 or AAV-Control as determined by ELISA (*n* = 10, One-way ANOVA with Tukey’s post hoc test, * *p* < 0.05). (**K**) The mRNA level of IL-1β, IL-6, and TNF-α in hippocampal samples from Control and CUMS mice infected with AAV-GLO1 or AAV-Control (*n* = 6, One-way ANOVA with Tukey’s post hoc test, * *p* < 0.05).

A specific adeno-associated virus (AAV) overexpressing GLO1 was constructed and microinjected into microglia in the hippocampus (Figure 6A). GFP expression confirmed infection of the hippocampal tissue (Figure 6B). The overexpression of GLO1 in the hippocampus following AAV-GLO1 infection was validated by qRT-PCR (Figure 6C). Subsequently, we assessed the effect of AAV-GLO1 on corticosterone levels in the hippocampus of mice. The results indicated that overexpression of GLO1 in the hippocampus alleviated the increase in corticosterone levels induced by CUMS (Figure 6D). Furthermore, the MWM and novel object recognition tests were employed to evaluate spatial learning and memory. AAV-GLO1 significantly reduced escape latency, increased the times of platform crossings, and enhanced the cognitive index in mice (Figure 6E–G). In addition, we measured the levels of MGO, AGEs, and RAGE in mouse serum and hippocampus, and the results showed that overexpression of GLO1 reduced MGO, AGEs, and RAGE levels in both the serum and hippocampus of CUMS mice (Figure 6H,I). These findings suggest that overexpression of GLO1 in the hippocampus can mitigate the toxic effects of AGEs and RAGE by reducing MGO levels. Moreover, we examined the changes in inflammatory factors in the hippocampus of mice. The results obtained from ELISA and qRT-PCR analyses revealed that the expression levels of inflammatory factors were markedly reduced in the group overexpressing GLO1 compared to the control group (Figure 6J,K). Based on these data, overexpression of GLO1 can partially clear MGO and, consequently, alleviate the inflammation cascade triggered by MGO-derived AGEs.

### 3.7. FPS-ZM1 Ameliorates CUMS-Induced Cognitive Impairment, Neuroinflammation, and MGO and Its Derivatives in Mice

**Figure 7:**
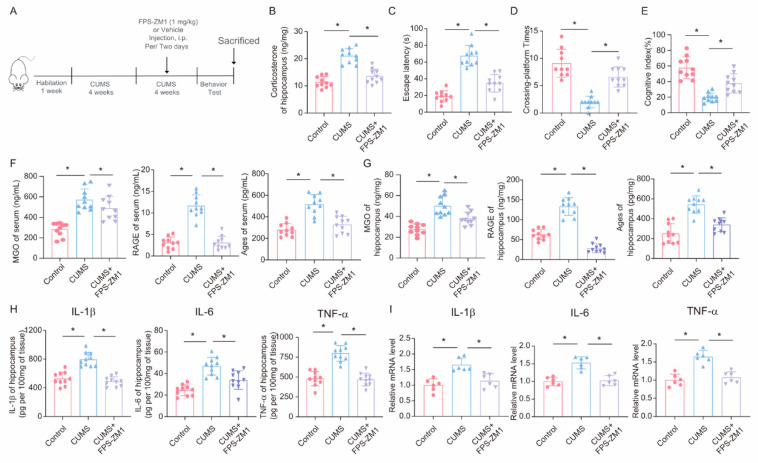
**FPS-ZM1 ameliorates CUMS-induced cognitive impairment, neuroinflammation, and MGO and its derivatives in mice.** (**A**) Schematic illustration of the experimental timeline for CUMS, FPS-ZM1 treatment, and behavioral testing. (**B**) Hippocampal corticosterone concentrations in Control, CUMS, and CUMS + FPS-ZM1 mice (*n* = 10; one-way ANOVA with Tukey’s post hoc test, * *p* < 0.05). (**C**,**D**) Escaping latency and Crossing-platform Times of Control, CUMS, and CUMS+FPS-ZM1 mice at the end of the CUMS procedure (*n* = 10, One-way ANOVA with Tukey’s post hoc test, * *p* < 0.05). (**E**) Cognitive Index of Control, CUMS, and CUMS+FPS-ZM1 mice at the end of the CUMS procedure (*n* = 10, One-way ANOVA with Tukey’s post hoc test, * *p* < 0.05). (**F**) Concentrations of MGO, RAGE, and Ages in the serum of Control, CUMS, and CUMS+FPS-ZM1 mice at the end of the CUMS procedure (*n* = 10, One-way ANOVA with Tukey’s post hoc test, * *p* < 0.05). (**G**) Concentrations of MGO, RAGE, and Ages in the hippocampus (*n* = 10, One-way ANOVA with Tukey’s post hoc test, * *p* < 0.05). (**H**) Levels of IL-1β, IL-6, and TNF-α in hippocampus lysates from Control, CUMS, and CUMS+FPS-ZM1 mice as determined by ELISA (*n* = 10, One-way ANOVA with Tukey’s post hoc test, * *p* < 0.05). (**I**) The mRNA level of IL-1β, IL-6, and TNF-α in hippocampal samples from Control, CUMS, and CUMS+FPS-ZM1 mice (*n* = 6, One-way ANOVA with Tukey’s post hoc test, * *p* < 0.05).

To further explore the role of the RAGE inhibitor FPS-ZM1 in CUMS-induced cognitive impairment and neuroinflammation, we injected the RAGE inhibitor FPS-ZM1 into CUMS mice to inhibit RAGE expression (Figure 7A). First, we assessed the effect of FPS-ZM1 on corticosterone levels in the hippocampus of mice. The results showed that intraperitoneal injection of FPS-ZM1 in mice could reduce the increase in corticosterone caused by CUMS (Figure 7B). Then, we conducted behavioral tests and found that, in the Morris Water Maze task, the escape latency of mice in the FPS-ZM1 injection group was significantly shortened compared to the CUMS group, and the times of platform crossings were significantly increased (Figure 7C,D). Meanwhile, the results of the novel object recognition test also showed that FPS-ZM1 injection ameliorated the cognitive index of CUMS mice (Figure 7E). Next, we measured the levels of MGO, AGEs, and RAGE in mouse serum and hippocampus using ELISA and found that FPS-ZM1 not only significantly inhibited RAGE expression but also reduced the production of MGO and AGEs to some extent (Figure 7F,G). We then measured the levels of inflammatory factors in hippocampal tissue lysates, and the results indicated that the RAGE inhibitor FPS-ZM1 alleviated neuroinflammation caused by chronic stress (Figure 7H,I). Overall, these experimental results suggest that the RAGE inhibitor FPS-ZM1 could ameliorate CUMS-induced cognitive impairment and neuroinflammation by blocking the binding of AGEs to RAGE.

## 4. Discussion

Methylglyoxal (MGO) is a byproduct of glycolysis, particularly formed through the non-enzymatic degradation of glyceraldehyde-3-phosphate and dihydroxyacetone phosphate [36]. On one hand, MGO itself exhibits potent cytotoxicity. It can directly damage cellular components via glycation, inducing oxidative stress and mitochondrial dysfunction. These effects further exacerbate the inflammatory environment in the brain, leading to neuronal damage, synaptic dysfunction, and subsequent behavioral abnormalities [37,38]. On the other hand, when MGO covalently modifies proteins, lipids, and nucleic acids to form advanced glycation end-products (AGEs), and these AGEs bind to the receptor for advanced glycation end-products (RAGE)—a multi-ligand receptor expressed on various cell types, including microglia and neurons—they activate intracellular signaling cascades, such as the nuclear factor κB (NF-κB) and mitogen-activated protein kinase (MAPK) pathways, both of which play crucial roles in regulating inflammatory responses [39,40]. Accumulating evidence suggests that the buildup of advanced glycation end products (AGEs) may play a role in the pathogenesis of neurodegenerative disorders, which are often characterized by neurotoxicity, peripheral neuropathies, and other neuronal complications. These disorders include Alzheimer’s disease (AD), multiple sclerosis (MS), and Parkinson’s disease (PD) [15,17,41,42]. In the context of stress induced by CUMS, increased production of MGO and AGEs is associated with the activation of multiple pro-inflammatory signaling pathways [16]. Therefore, addressing the effects of MGO-AGEs may help raise awareness of potential toxicities resulting from exposure to endogenous or dietary AGEs. Additionally, MGO-mediated AGEs, formed in cells driven by glycolysis, are actively involved in MS lesions. Other studies have reported that MGO exposure leads to mitochondrial dysfunction and the activation of mitophagy [43]. However, the role of MGO-AGEs in neuroinflammation caused by chronic stress has not yet been reported.

Studies have reported that chronic stress is associated with an increase in glycolytic flux, which enhances the production of MGO. Hyperactivation of the hypothalamic–pituitary–adrenal (HPA) axis induced by stress leads to elevated corticosterone levels, which, in turn, stimulates gluconeogenesis and glycolysis in peripheral tissues and the brain. This metabolic shift can increase the levels of the reactive glycolytic byproduct MGO [7,38]. In this study, we demonstrated an increase in endogenous toxic metabolites MGO and its derivatives under conditions of chronic stress-induced cognitive dysfunction and neuroinflammation. Moreover, chronic stress downregulated the expression of glyoxalase 1 (GLO1) in the hippocampus of mice, potentially impairing the detoxification of MGO. Additionally, we treated microglia with the stress hormone corticosterone and found that corticosterone increased the levels of MGO and its derivatives in microglia, accompanied by an inflammatory response.

Currently, several clinical strategies can block the toxic effects of MGO or its derivatives. MGO scavengers, such as metformin and N-acetylcysteine (NAC), have been shown to reduce MGO levels and prevent the formation of AGEs. NAC, a precursor to glutathione, acts as an antioxidant and reactive oxygen species (ROS) scavenger, reducing oxidative stress and inflammation. While both compounds have shown efficacy in reducing neuroinflammatory markers and ameliorating behavioral outcomes in preclinical models, they still contain many limitations. For example, the ameliorative effects of metformin on neuroinflammation may vary across disease models, and its ameliorative effects on memory recovery and oxidative stress in AD models have been inconsistent across acute and chronic administrations. In addition, metformin may cause side effects such as gastrointestinal discomfort, which limits its use in long-term treatment. The antioxidant capacity of NAC may not be sufficient to completely counteract oxidative stress in neuroinflammation, resulting in limited long-term effects on neurological functional recovery. Moreover, the low bioavailability of NAC may affect its central nervous system drug concentration [44,45]. Additionally, MGO levels can be reduced by targeting the MGO detoxification system, specifically the glyoxalase system [9,46]. In this study, we opted for the overexpression of GLO1 to detoxify MGO. The results showed that after 8 weeks of CUMS, mice overexpressing GLO1 exhibited significantly lower levels of MGO and its derivatives, as well as ameliorated cognitive function and reduced neuroinflammation compared to the control group. Although our results suggest that the mice in the MWM escape latency decreased and increased the times across the platform. However, we did not observe significant differences in search strategies in mice in this study. This provides a new direction for our next research and helps us to understand more deeply how intervention with MGO improves the cognitive function impairment of mice during chronic stress.

Another promising therapeutic strategy is to inhibit the RAGE pathway, thereby blocking the pro-inflammatory effects of MGO-derived AGEs. Small-molecule inhibitors of RAGE or antibodies targeting RAGE have been shown to reduce neuroinflammatory responses in preclinical models [24,26,47]. In this study, we administered the RAGE inhibitor FPS-ZM1 to animals in the CUMS model and found that RAGE inhibition also ameliorated CUMS-induced cognitive dysfunction and neuroinflammation. These results indicate that reducing MGO and its derivatives may represent a novel approach for mitigating neuroinflammation caused by chronic stress. Despite the therapeutic potential of targeting MGO in CUMS-induced neuroinflammation, several challenges remain. A key challenge is the precise regulation of MGO levels, as complete depletion of MGO could interfere with normal cellular processes. Therefore, developing therapeutic strategies that selectively target pathological levels of MGO while preserving its physiological functions is essential.

It is well known that the CUMS model is a classical model for studying cognitive function and emotional abnormalities caused by depressive-like behaviors. Anhedonia and helplessness can be observed in behavioral experiments such as the sucrose preference test (SPT) and the forced swimming test (FST). In the MWM and novel object recognition experiments, a decline in cognitive function was observed in mice [48]. Cognitive and emotional dysfunction induced by the CUMS model involves a variety of neurobiological mechanisms, and the CUMS model has been implicated in cognitive and emotional dysfunction; these include neurotransmitter imbalance (e.g., 5-hydroxytryptamine and noradrenaline), neuroinflammation, decreased expression of Neurotrophin (e.g., BDNF), and metabolic abnormalities [48,49]. These mechanisms play an important role in both cognitive and emotional dysfunction. Systemic low-grade inflammation caused by CUMS is a common feature of chronic metabolic disorders, which have also been found to drive neuroinflammation and lead to neurodegenerative changes. Scientists coined the terms “metaflammation” and “immunometabolism” to describe the tight relationship between the two [50,51,52,53]. In a previous study, we found that CUMS can lead to a high glycolytic response in the hippocampus of mice and that high glucose in the hippocampus of mice leads to the occurrence of neuroinflammation, while mice develop abnormal cognitive functions. We continue previous studies to examine CUMS-induced neuroinflammation and cognitive impairment in terms of glucose metabolism disorders. In the present study, we mainly focused on whether the intervention of MGO, an intermediate product of glycolysis, could improve the neuroinflammation and cognitive impairment caused by CUMS. At the same time, we also know CUMS on emotional dysfunction, but in this study, we mainly focus on and investigate the intervention MGO on CUMS-induced neuroinflammation and cognitive function. In the following study, we continue to focus on the regulatory effect of intervening glucose metabolism on mood disorders in CUMS mice.

## 5. Conclusions

MGO is a highly reactive dicarbonyl compound and a byproduct of glycolysis. It can trigger neuroinflammation by activating microglia inflammatory responses and increasing oxidative stress and inflammatory cascades in the brain. In this study, we found that chronic unpredictable mild stress (CUMS) leads to high levels of MGO and its derivatives in the hippocampus of mice, and they have the potential to increase the release of pro-inflammatory factors by increasing ROS content, which may be related to the effects of CUMS, activation of MAPK signaling pathway promotes the occurrence and development of neuroinflammation. Interestingly, by reducing the levels of MGO or its derivatives, we were able to alleviate CUMS-induced neuroinflammation and cognitive dysfunction. This suggests that targeted clearance of MGO, or inhibition of its derivative RAGE, may be a promising therapeutic strategy to alleviate CUMS and stress-related psychological disorders.

## Data Availability

The datasets generated during and/or analyzed during the current study are available from the corresponding author upon reasonable request. The data are not publicly available due to specific ethical and privacy considerations.

## Supplementary Materials

The following supporting information can be downloaded at: https://www.mdpi.com/article/10.3390/cells14060397/s1, Table S1: Sequences of qPCR primers.

## Abbreviations

Corticosterone (Cort), Hypothalamic–Pituitary–Adrenal (HPA), Glucocorticoids (GC), Reactive Oxygen Species (ROS), CUMS (Chronic Unpredictable Mild Stress), Advanced Glycation End Products (AGEs), Methylglyoxal (MGO), Glyoxalase 1 (GLO1), Advanced Glycation End Products (RAGE), Chronic Unpredictable Mild Stress (CUMS), Adeno-Associated Virus (AAV), Nuclear Factor κB (NF-κB), Mitogen-Activated Protein Kinase (MAPK), Alzheimer’s Disease (AD), Multiple Sclerosis (MS), Parkinson’s Disease (PD), N-Acetylcysteine (NAC).
