# Supplementary material for: Depletion of MGO or Its Derivatives Ameliorate CUMS-Induced Neuroinflammation
Source: Cells. 2025 Mar 8;14(6):397. doi: 10.3390/cells14060397 (PMC11941696; doi:10.3390/cells14060397)
Supplement: Supplementary file 1 [file cells-14-00397-s001.zip › Supplementary Table s1 Sequences of qPCR primers.pdf]

Supplementary Table S1 Sequences of qPCR primers

Gene name	Forward	Reverse
GLO1	GGGGCACTGAAGATGACGAG	AAGAGGCAGGCAGCAGCACA
RAGE	ACATGTGTGTCTGAGGGAAGC	AGCTCTGACCGCAGTGTAAAG
IL-1β	CAGGCAGGCAGTATCACTCATTG	GCTTTTTTGTTGTTCATCTCGGA
IL-6	TAGTCCTTCCTACCCCAATTTCC	TTGGTCCTTAGCCACTCCTTC
TNF-α	ACGGCATGGATCTCAAA	AGATAGCAAATCGGCTGAC
β-actin	GGCTGTATTCCCCTCCATCG	CCAGTTGGTAACAATGCCATGT
