# Supplementary figures and images for: Depletion of MGO or Its Derivatives Ameliorate CUMS-Induced Neuroinflammation
Source: Cells. 2025 Mar 8;14(6):397. doi: 10.3390/cells14060397 (PMC11941696; doi:10.3390/cells14060397)

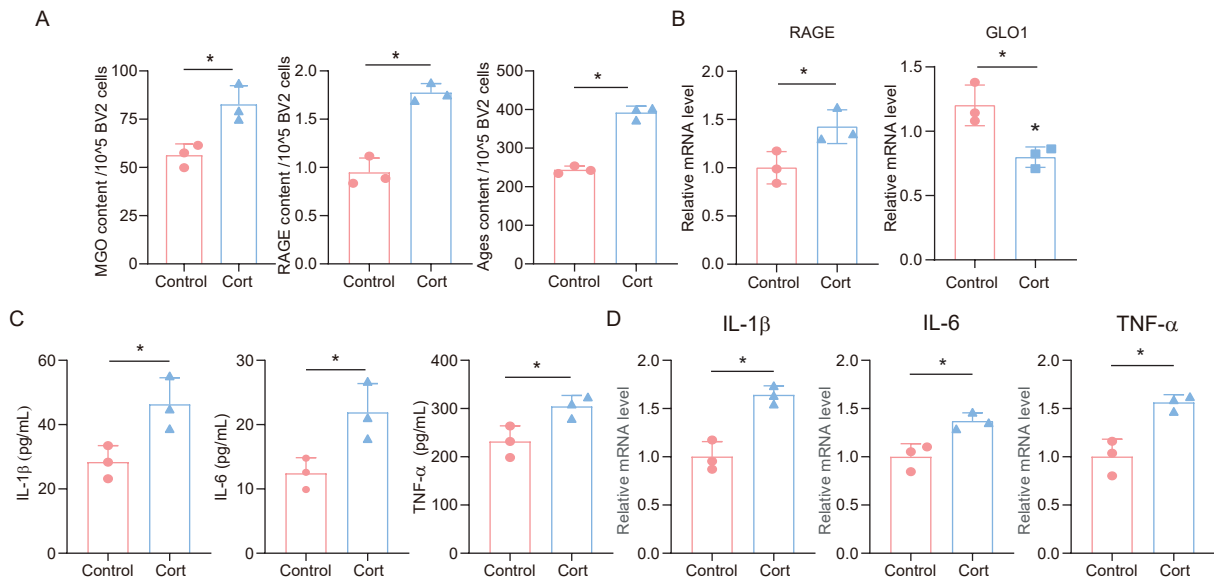

Figure S1

Supplement: Supplementary file 1 [file cells-14-00397-s001.zip › FIgure S1.pdf]
